# Comparisons of the segments of left-sided double-lumen tracheobronchial tubes as industrial products

**DOI:** 10.1186/s12871-022-01698-2

**Published:** 2022-06-08

**Authors:** Zen’ichiro Wajima, Toshiya Shiga, Kazuyuki Imanaga

**Affiliations:** 1 Department of Anesthesia, Seijin Hospital, 3-2-1 Shimane, Adachi-ku, Tokyo, 121-0815 Japan; 2 Department of Anesthesiology, Saitama Medical University Hospital, Moroyama, Saitama 350-0495 Japan; 3 Department of Anesthesiology and Intensive Care Medicine, School of Medicine, International University of Health and Welfare, Chiba, 286-8686 Japan; 4 Department of Anesthesia, Shonan Kamakura General Hospital, Kanagawa, 247-8533 Japan

**Keywords:** Left-sided double-lumen tracheobronchial tubes, Segment, Quality, Feature, Aspect, Margin of safety, Industrial products

## Abstract

**Background:**

Although there are at least seven manufacturers producing left-sided double-lumen tubes (DLTs), there have been few reports comparing the segments of these DLTs. In this study, we compared various segments of left-sided DLTs further.

**Materials and methods:**

We examined five manufacturers’ left-sided DLTs: Mallinckrodt, Portex, Rüsch, Sheridan, and Daiken-medical. We conducted the following six trials or measurements, and three supplemental trials or measurements: First, we tried to pass various sizes of steel balls down each lumen in order from the smallest (3 mm) to largest (4.5 mm). If the ball passed on the first attempt, we tried just once; otherwise, we made a second attempt. Second, we measured the external diameter of tracheal and bronchial cuff using a profile projector. Third, we measured the length of the cuff and tip of the bronchial segment of the tubes using the profile projector. Fourth, we measured various lengths of the tubes. Fifth, we measured the external diameter of both lumens and the tubules for tracheal and bronchial cuff inflation. Finally, we measured various cross-sectional areas including the tracheal lumen, bronchial lumen, and tubules for cuff inflation. We also conducted three supplemental studies. First, we measured air volume in the cuff when intracuff pressure equaled 2 or 3 kPa. Second, we defined the configuration of the tracheal and bronchial cuffs. Third, we defined the presence or absence of bevels and also measured the angle of the bevels using the profile projector.

**Results:**

We performed nine trials and measurements and found large disparities between each manufacturer’s tubes.

**Conclusions:**

The large disparities found between the measurements of the five manufacturers’ tubes may be due to different lots or changes in specifications made by each manufacturer. We found tubes exhibiting lower quality, such as deformations, and non-universal and inconsistent sizing, in the comparison of the manufacturers’ tubes. Practitioners should be aware of the features and aspects of these tubes.

**Supplementary Information:**

The online version contains supplementary material available at 10.1186/s12871-022-01698-2.

## Background

Although at least seven manufacturers are producing left-sided double-lumen tracheobronchial tubes (DLTs) [1–3], which are used for lung separation and one-lung ventilation [4], few reports have compared the segments of these DLTs. Benumof et al. [5] defined the “margin of safety” in positioning a DLT as the length of the tracheobronchial tree over which the DLT may be moved or positioned without obstructing a conducting airway and measured the margin of safety in positioning three manufacturers’ DLTs available in 1987. However, they did not measure the tubes themselves but only the lengths of the right and left mainstem bronchi with in vivo fiberoptic bronchoscopy and in fresh cadavers and lung casts [2]. In 1996, Watterson and Harrison [1] compared a range of available left-sided DLTs to show the differences between them with respect to the length of the endobronchial segment because a tube with a short endobronchial segment may be better suited to anesthesia under the conditions imposed by double sequential lung transplantation. Later, in 2006, Partridge and Russell [2] measured the actual lengths of the bronchial cuff and bronchial tip on DLTs from four manufacturers to provide tube dimensions for the margin of safety.

However, there have been few measurements and/or investigations of air volume in the cuffs, angles of bevels, the transverse and longitudinal external diameters of the tracheal and bronchial cuffs after cuff inflation, various lengths of different tubes, external diameters of both lumens and the tubules (air channels, inflation lumen [6]) for tracheal and bronchial cuff inflation, and various cross-sectional areas of tracheal and bronchial lumens and tubules for cuff inflation with area measurement software [3]. We thought it beneficial to measure these factors in various DLTs which are industrial products in greater detail. Therefore, under the hypothesis that there would be large disparities between each manufacturer’s tubes, inaccurate dimensions, and potential disadvantages, we aimed to measure and investigate various DLT configurations in this study.

## Materials and methods

We examined left-sided DLTs (35 and 37 Fr; two different lots of each) from five manufacturers that we obtained in January 2017 (product name in parentheses): Mallinckrodt (Bronch-Cath™), Portex (Blue Line™), Rüsch (Bronchopart®), Sheridan (SHER-I-BRONCH®), and Daiken Medical (Coopdech) (Table 1).

**Table 1:** Double lumen tube product name, manufacturer, geographic origin of manufacturer, and distributor in Japan

Product name	Manufacturer	Geographic Origin of Manufacturers	Distributor in Japan
Bronch-Cath™	Mallinckrodt	Tullamore, Ireland	Covidien
Blue Line™	Portex	Hythe, UK	Smith Medical
Bronchopart®	Rüsch	Athlone, Ireland	Toray Medical
SHER-I-BRONCH®	Sheridan	Athlone, Ireland	Teleflex
Coopdech	Daiken Medical	Osaka, Japan	Daiken Medical

We conducted the following six trials or measurements, and three supplemental trials or measurements: 1. investigation of the passage of steel balls of various diameters through each lumen, 2. measurement of the external diameter of the tracheal and bronchial cuff after cuff inflation, 3. measurement of the lengths of the cuffs and tip of the bronchial segment of the tubes [5], 4. measurement of various lengths of the tubes, 5. measurement of the external diameter of both lumens and the tubules for tracheal and bronchial cuff inflation, and 6. measurement of various cross-sectional areas; and Suppl. 1. measurement of air volume in the cuffs when intracuff pressure equals 2 and 3 kPa, Suppl. 2. categorization of the tracheal and bronchial cuff configurations, and Suppl. 3. investigation of the presence or absence of a bevel [1] and measurement of the bevel angle. All measurements were performed with the pre-loaded intubation stylet removed from the DLT. The supplemental studies’ methods with results and discussion are shown in “Additional files 1, 2 and 3”.

All average diameters and lengths were calculated with the use of Microsoft Excel.

### Passage of steel balls of various sizes

We investigated whether steel balls of various diameters (3, 3.5, 4, and 4.5 mm) would pass through each lumen (from the limb opening of the tube to the tracheal or bronchial lumen outlet) in order from smallest to largest by gravity. Neither the DLTs nor the steel balls were lubricated. Two attempts were made unless the ball passed on the first attempt.

### Measurement of the external diameters of tracheal and bronchial cuffs

We measured the external diameters of the tracheal and bronchial cuffs (transverse and longitudinal) (internal cuff pressure: 2.0 ± 0.1 kPa [≒ 20 cmH_2_O, 15 mmHg] [ISO 5361]) with each tube on the profile projector.

We calculated the average of the transverse and longitudinal external diameters of two tubes each of the two French sizes obtained from each manufacturer. Moreover, we calculated both the average of the transverse external diameters and that of the longitudinal external diameters of the tubes, all of which were obtained from different lots. Finally, we calculated the average of the four diameters obtained from the measurements.

### Measurement of length of the cuff and tip of the bronchial segment of the tubes

**Fig. 1:**
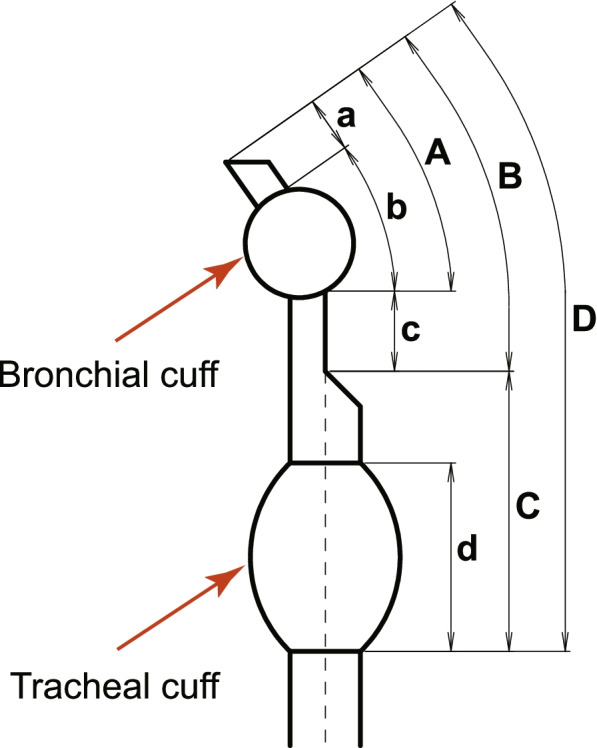
Measurement of length of the cuff and tip of the bronchial segment of the tubes. We measured the length of the cuffs and tips of the bronchial segment of the tubes after we set the intracuff pressure equal to 2.0 ± 0.1 kPa (about 20 cmH_2_O). One of the most important bronchial segments is “A (a+b)” because it plays a major part in the “margin of safety”

We measured the length of the cuffs and tips of the bronchial segment of the tubes after we set the intracuff pressure to 2.0 ± 0.1 kPa (about 20 cmH_2_O) with the tubes on the profile projector (Fig. 1). Because the inflated cuffs were not symmetrical, we used the maximum value measured.

For each manufacturer’s tubes, we calculated the average of each length on two tubes each of the two French sizes. All tubes were obtained from different lots.

### Measurement of various lengths of the tubes

**Fig. 2:**
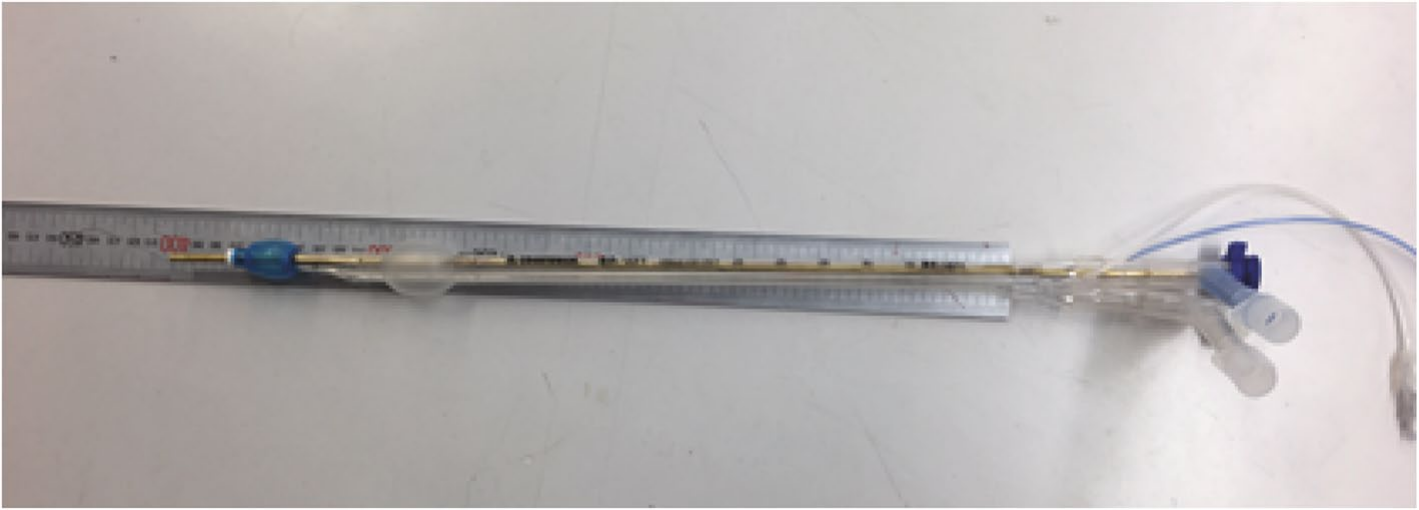
Measurement of various lengths of the tubes and presence or absence of bevels and measuring angle of bevels. We inserted a brazen rod (4 mm in diameter) into each tube to straighten it to measure the various tube lengths

We measured four different lengths of the tubes: I. the distance between the bronchial lumen tip (patient end) and the tip of the pilot balloon/inflation valve (the longest length); II. the distance between the bronchial lumen tip (patient end) and the bronchoscope port; III. the distance between the bronchial lumen tip (patient end) and the slip joint (a tracheal tube connector) [7] (except on the Portex and Daiken Medical tubes, which do not have a structural slip joint); and IV. the distance between the bronchial lumen tip (patient end) and the Y-shaped connector (“Y” connector [8]) (patient side) [1]. We inserted a brazen rod (4 mm in diameter) into each tube to straighten it to measure the various lengths (Fig. 2).

### Measurement of the external diameters of both lumens and the tracheal and bronchial cuff inflation tubules

**Fig. 3:**
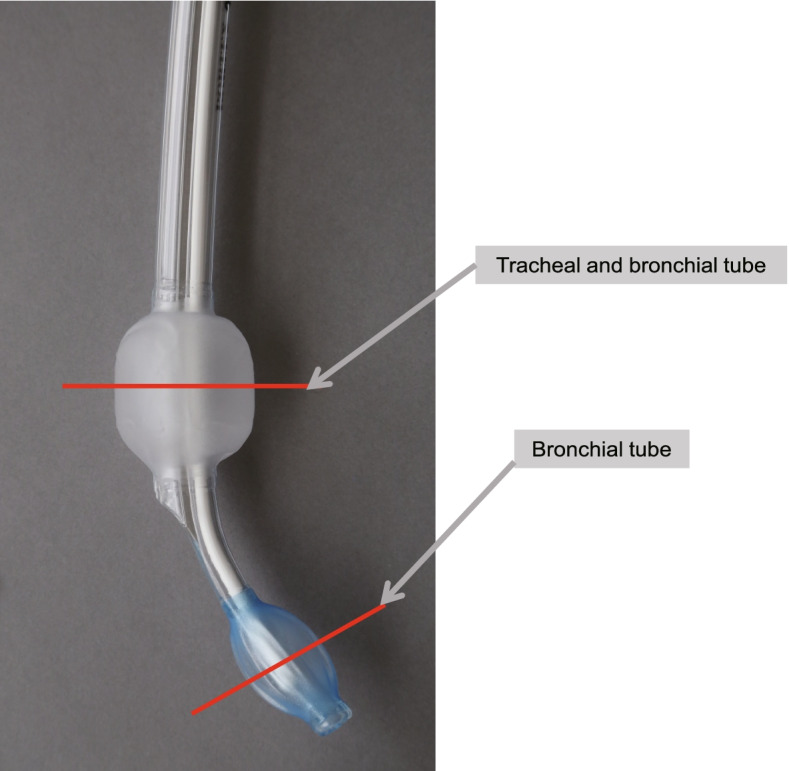
Measurement of the external diameters of both lumens and the tracheal and bronchial cuff inflation tubules, and measurement of various cross-sectional areas. We cut the tubes across their diameter at the center of the cuff location (indicated by the red lines) and detached the cuffs

**Fig. 4:**
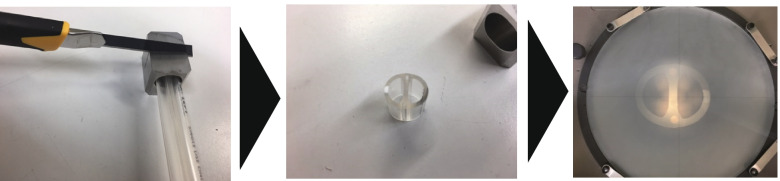
Measuring road map. The left-hand panel shows the tube being cut. The middle panel shows the cut tube. We measured the external diameters (long and short axes) of both lumen parts, the transverse and longitudinal diameters of the bronchial lumen, and the tubules for tracheal and bronchial cuff inflation on the profile projector (the right-hand panel)

**Fig. 5:**
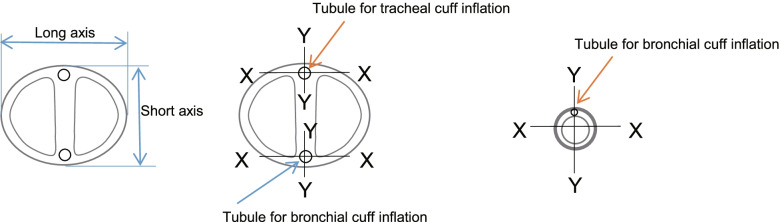
Measurement of the external diameters of both lumens and the tracheal and bronchial cuff inflation tubules. We measured the external diameters (long and short axes) of both lumen parts, the transverse and longitudinal diameters of the bronchial lumen, and the tubules for tracheal and bronchial cuff inflation (X-X and Y-Y)

First, we cut all tubes crosswise at the center point of the cuff location (Figs. 3, 4). Then, after detaching the cuff, we measured the external diameters (long and short axes) of both lumens, the transverse and longitudinal diameters of the bronchial lumen, and the tubules for tracheal and bronchial cuff inflation on the profile projector (Figs. 4 and 5).

We calculated the average of each length (long and short and/or transverse and longitudinal) of two tubes each of the two French sizes obtained from each manufacturer. Moreover, we calculated the average of the long external diameters and short external diameters and that of the transverse external diameters and longitudinal external diameters of the tubes, all which were obtained from different lots. Finally, we calculated the average of the four lengths obtained from the measurements.

### Measurement of various cross-sectional areas

We measured the cross-sectional areas of the tracheal lumens, bronchial lumens, tubules for tracheal cuff inflation, and tubules for bronchial cuff inflation of both lumens, and also those of the bronchial lumens and tubules for bronchial cuff inflation of the bronchial lumen. After cutting the tubes, we measured all areas with the area measurement software of a Keyence Digital Microscope VHX-1000 (20 ×) (Keyence Corporation, Osaka, Japan) (Figs. 3, 4).

## Results

### Passage of steel balls of various sizes

One Bronchopart® 35 Fr tube showed different results for the first and second attempts (tracheal lumen: 3.5 mm). Furthermore, different results were obtained in different lots of the Blue Line™ (35 Fr; bronchial lumen: 4.0 mm), Bronchopart® (35 Fr; tracheal lumen: 3.5 mm; bronchial lumen: 4.0 mm), SHER-I-BRONCH® (37 Fr; tracheal lumen: 4.5 mm), and Bronch-Cath™ (37 Fr; bronchial lumen: 4.5 mm) (Table 2).

**Table 2:** Passage of steel balls of various sizes

Product name	35 Fr										37 Fr									
	**Sample number**	**Lot No**	**Tracheal lumen**				**Bronchial lumen**				**Sample number**	**Lot No**	**Tracheal lumen**				**Bronchial lumen**			
			**3.0 mm**	**3.5 mm**	**4.0 mm**	**4.5 mm**	**3.0 mm**	**3.5 mm**	**4.0 mm**	**4.5 mm**			**3.0 mm**	**3.5 mm**	**4.0 mm**	**4.5 mm**	**3.0 mm**	**3.5 mm**	**4.0 mm**	**4.5 mm**
Bronch-Cath™	①	201210608X	〇	〇	〇	× ×	〇	〇	〇	× ×	⑪	201411237X	〇	〇	〇	〇	〇	〇	〇	〇
	②	201503168X	〇	〇	〇	× ×	〇	〇	〇	× ×	⑫	201410348X	〇	〇	〇	〇	〇	〇	〇	× ×
Blue Line™	③	3227850	〇	〇	〇	× ×	〇	〇	× ×	× ×	⑬	3227862	〇	〇	〇	× ×	〇	〇	〇	× ×
	④	3227851	〇	〇	〇	× ×	〇	〇	〇	× ×	⑭	3227865	〇	〇	〇	× ×	〇	〇	〇	× ×
Bronchopart®	⑤	15GE29J	〇	× ×	× ×	× ×	〇	〇	× ×	× ×	⑮	16HE33J	〇	〇	〇	× ×	〇	〇	〇	× ×
	⑥	15DE16J	〇	× 〇	× ×	× ×	〇	〇	〇	× ×	⑯	15GE29J	〇	〇	〇	× ×	〇	〇	〇	× ×
SHER-I-BRONCH®	⑦	73L1500302	〇	〇	〇	× ×	〇	〇	〇	〇	⑰	73L1500369	〇	〇	〇	〇	〇	〇	〇	〇
	⑧	73L1500168	〇	〇	〇	× ×	〇	〇	〇	〇	⑱	73K1600076	〇	〇	〇	× ×	〇	〇	〇	〇
Coopdech	⑨	B161013X	〇	〇	〇	× ×	〇	〇	〇	× ×	⑲	B161205X	〇	〇	〇	〇	〇	〇	〇	〇
	⑩	B161017X	〇	〇	〇	× ×	〇	〇	〇	× ×	⑳	B161209X	〇	〇	〇	〇	〇	〇	〇	〇

Passed: ○, Did not pass: ✕

In the 35 Fr tube tracheal lumens, 4.0-mm diameter steel balls passed through all lumens except the Bronchopart® lumen. In the 35 Fr tube bronchial lumens, 4.5-mm diameter steel balls could pass only through the SHER-I-BRONCH® lumen. In the 37 Fr tube tracheal lumens, 4.5-mm diameter steel balls could pass through the Bronch-Cath™ and Coopdech lumens. In the 37 Fr tube bronchial lumens, 4.5-mm diameter steel balls could pass through the SHER-I-BRONCH® and Coopdech lumens (Table 2).

### Measurement of the external diameters of the tracheal and bronchial cuffs

Among the 35 and 37 Fr tubes, we found large disparities in the external diameters of the tracheal and bronchial cuffs between each manufacturer’s tubes (Table 3).

**Table 3:** Measurement of the external diameters of tracheal and bronchial cuffs (Unit: mm)

	**35Fr**								**37Fr**							
	**Sample Number**	**Lot No**	**Tracheal Cuff**			**Bronchial Cuff**			**Sample Number**	**Lot No**	**Tracheal Cuff**			**Bronchial Cuff**		
**Product Name**			**X-X**	**Y**_**1**_**-Y**_**1**_	**Mean**	**X-X**	**Y**_**2**_**-Y**_**2**_	**Mean**			**X-X**	**Y**_**1**_**-Y**_**1**_	**Mean**	**X-X**	**Y**_**2**_**-Y**_**2**_	**Mean**
Bronch-Cath™	①	201210608X	26.2	24.8	25.50	20.4	21.0	20.7	⑪	201411237X	27.1	25.6	26.4	20.7	20.9	20.8
	②	201503168X	25.7	24.6	25.15	20.5	21.0	20.8	⑫	201410348X	27.5	25.4	26.5	20.9	21.3	21.1
		Mean	26.0	24.7	25.33	20.5	21.0	20.73		Mean	27.3	25.5	26.40	20.8	21.1	20.95
Blue Line™	③	3227850	30.0	30.1	30.05	19.8	21.0	20.4	⑬	3227862	32.7	32.6	32.7	19.6	20.7	20.2
	④	3227851	29.5	29.2	29.35	19.9	20.9	20.4	⑭	3227865	32.0	32.4	32.2	19.0	19.9	19.5
		Mean	29.8	29.7	29.70	19.9	21.0	20.40		Mean	32.4	32.5	32.43	19.3	20.3	19.80
Bronchopart®	⑤	15GE29J	29.4	28.8	29.10	14.4	15.0	14.7	⑮	16HE33J	29.0	28.9	29.0	14.9	14.8	14.9
	⑥	15DE16J	28.1	28.0	28.05	14.4	14.7	14.6	⑯	15GE29J	29.2	28.2	28.7	12.4	12.9	12.7
		mean	28.8	28.4	28.58	14.4	14.9	14.63		Mean	29.1	28.6	28.83	13.7	13.9	13.75
SHER-I-BRONCH®	⑦	73L1500302	24.1	23.1	23.60	19.1	19.6	19.4	⑰	73L1500369	27.6	25.6	26.6	18.3	20.1	19.2
	⑧	73L1500168	22.8	22.9	22.85	17.5	18.3	17.9	⑱	73K1600076	26.3	26.6	26.5	19.1	19.7	19.4
		Mean	23.5	23.0	23.23	18.3	19.0	18.63		Mean	27.0	26.1	26.53	18.7	19.9	19.30
Coopdech	⑨	B161013X	24.4	25.0	24.70	17.5	17.8	17.7	⑲	B161205X	24.7	25.1	24.9	18.1	17.7	17.9
	⑩	B161017X	24.1	25.1	24.60	18.0	18.0	18.0	⑳	B161209X	24.4	25.0	24.7	18.0	17.4	17.7
		Mean	24.3	25.1	24.65	17.8	17.9	17.83		Mean	24.6	25.1	24.80	18.1	17.6	17.80

X-X: transverse, Y_1_-Y_1_ and Y_2_-Y_2_: longitudinal

### Measurement of length of the cuff and tip of the bronchial segment of the tubes

The sum of “A”, the length of the bronchial end (patient end) (a) and bronchial cuff length (b), in order (from longest to shortest) in the 35 Fr tubes was Bronch-Cath™ > SHER-I-BRONCH® > Blue Line™ > Coopdech > Bronchopart®, and that in the 37 Fr tubes in order was Blue Line™ > Coopdech > SHER-I-BRONCH® > Bronch-Cath™ > Bronchopart® (Fig. 1, Table 4).

**Table 4:** Measurement of length of the cuff and tip of the bronchial segment of the tubes (Unit: mm)

Product Name	35Fr										37Fr									
	**Sample Number**	**Lot No**	**a**	**b**	**c**	**d**	**A (= a + b)**	**B (= a + b + c)**	**C**	**D (= B + C)**	**Sample Number**	**Lot No**	**a**	**b**	**c**	**d**	**A (= a + b)**	**B (= a + b + c)**	**C**	**D (= B + C)**
Bronch-Cath™	①	201210608X	14.3	19.3	23.6	44.5	33.6	57.2	52.2	109.4	⑪	201411237X	9.0	19.3	27.8	45.8	28.3	56.1	55.1	111.2
	②	201503168X	12.1	19.3	25.9	41.8	31.4	57.3	51.8	109.1	⑫	201410348X	10.8	19.2	28.0	43.1	30.0	58.0	52.7	110.7
		Mean	13.20	19.30	24.75	43.15	32.50	57.25	52.00	109.25		Mean	9.90	19.25	27.90	44.45	29.15	57.05	53.90	110.95
Blue Line™	③	3227850	4.1	27.2	30.7	31.5	31.3	62.0	38.0	100.0	⑬	3227862	4.3	27.6	29.5	39.2	31.9	61.4	45.9	107.3
	④	3227851	5.0	26.6	30.7	31.8	31.6	62.3	36.1	98.4	⑭	3227865	5.2	28.2	28.9	38.3	33.4	62.3	43.6	105.9
		Mean	4.55	26.90	30.70	31.65	31.45	62.15	37.05	99.20		Mean	4.75	27.90	29.20	38.75	32.65	61.85	44.75	106.60
Bronchopart®	⑤	15GE29J	6.3	15.7	23.1	41.5	22.0	45.1	50.0	95.1	⑮	16HE33J	7.3	15.2	25.1	42.8	22.5	47.6	51.3	98.9
	⑥	15DE16J	5.0	17.9	21.5	40.7	22.9	44.4	51.0	95.4	⑯	15GE29J	7.2	15.2	27.3	40.8	22.4	49.7	49.8	99.5
		Mean	5.65	16.80	22.30	41.10	22.45	44.75	50.50	95.25		Mean	7.25	15.20	26.20	41.80	22.45	48.65	50.55	99.20
SHER-I-BRONCH®	⑦	73L1500302	8.9	23.7	28.2	40.1	32.6	60.8	48.8	109.6	⑰	73L1500369	6.4	24.8	30.7	36.1	31.2	61.9	48.4	110.3
	⑧	73L1500168	6.9	23.9	29.6	40.4	30.8	60.4	49.0	109.4	⑱	73K1600076	6.7	23.4	30.1	38.5	30.1	60.2	46.7	106.9
		Mean	7.90	23.80	28.90	40.25	31.70	60.60	48.90	109.50		Mean	6.55	24.10	30.40	37.30	30.65	61.05	47.55	108.60
Coopdech	⑨	B161013X	11.5	18.2	29.4	34.8	29.7	59.1	47.8	106.9	⑲	B161205X	11.8	20.5	27.3	34.5	32.3	59.6	46.6	106.2
	⑩	B161017X	12.0	19.2	27.8	35.4	31.2	59.0	47.9	106.9	⑳	B161209X	12.2	19.3	28.6	34.1	31.5	60.1	47.2	107.3
		Mean	11.75	18.70	28.60	35.10	30.45	59.05	47.85	106.90		Mean	12.00	19.90	27.95	34.30	31.90	59.85	46.90	106.75

### Measurement of various lengths of the tubes

Results of measurements of the four distances I, II, III, and IV are detailed in Table 5.

**Table 5:** Measurement of various lengths of the tubes (Unit: mm; N/A: not applicable)

Product Name	35 Fr						37 Fr						
	Sample Number	Lot No	I	II	III	IV	Sample Number	Lot No	I	II	III	IV	
Bronch-Cath™	①	201210608X	541	480	438	322	⑪	201411237X	542	477	435	318	
	②	201503168X	538	478	435	318	⑫	201410348X	549	484	442	322	
		mean	539.5	479.0	436.5	320.0		mean	545.5	480.5	438.5	320.0	
Blue Line™	③	3227850	620	445		397	⑬	3227862	615	441		395	
	④	3227851	619	447		400	⑭	3227865	621	445		399	
		mean	619.5	446	N/A	398.5		mean	618.0	443.0	N/A	397.0	
Bronchopart®	⑤	15GE29J	530	445	399	295	⑮	16HE33J	533	450	400	297	
	⑥	15DE16J	535	445	395	295	⑯	15GE29J	539	453	404	302	
		mean	532.5	445.0	397.0	295.0		mean	536.0	451.5	402.0	299.5	
SHER-I-BRONCH®	⑦	73L1500302	583	507	459	355	⑰	73L1500369	577	504	455	353	
	⑧	73L1500168	581	506	457	355	⑱	73K1600076	579	505	455	352	
		mean	582.0	506.5	458.0	355.0		mean	578.0	504.5	455.0	352.5	
Coopdech	⑨	B161013X	653	476		369	⑲	B161205X	650	473		367	
	⑩	B161017X	656	477		370	⑳	B161209X	650	473		368	
		mean	654.5	476.5	N/A	369.5		mean	650.0	473.0	N/A	367.5	

I. Distance between the bronchial lumen tip (patient end) and the tip of the pilot balloon/inflation valve (the longest length)

II. Distance between the bronchial lumen tip (patient end) and bronchoscope port

III. Distance between the bronchial lumen tip (patient end) and slip joint (however, we did not measure the distance on Portex and Daiken-medical because they do not have structurally slip joint)

IV. Distance between the bronchial lumen tip (patient end) and the Y-shaped connector

### Measurement of the external diameters of both lumens and the tracheal and bronchial cuff inflation tubules

**Fig. 6:**
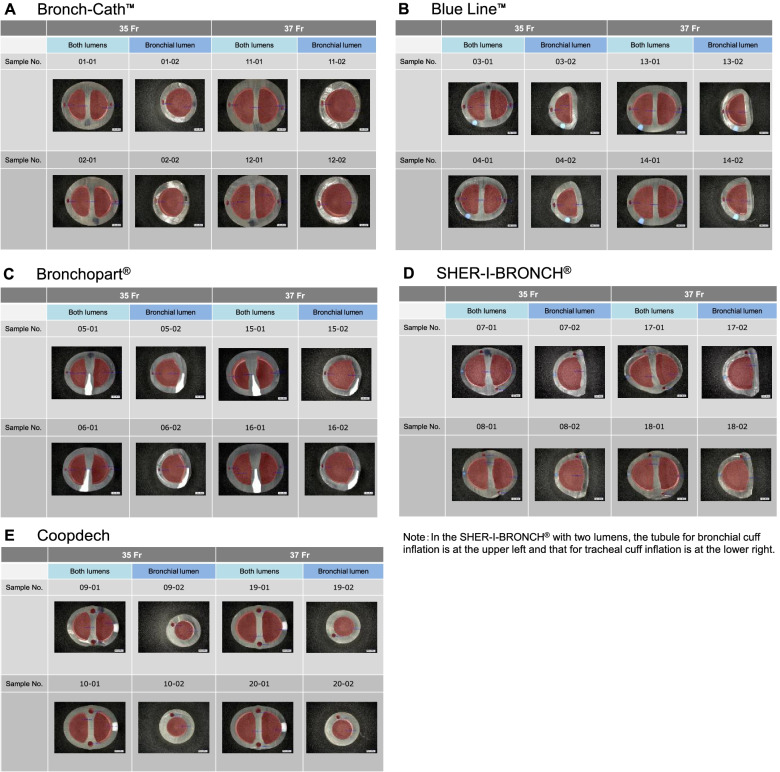
Cross-sectional views of each tube. We found large disparities between each manufacturer’s tubes. Both lumen parts in all tubes were longer in the horizontal measurement

The results of external diameters (long and short axes) of both lumens and the transverse and longitudinal diameters of the bronchial lumens, and the transverse and longitudinal diameters of the tubules for tracheal and bronchial cuff inflation for both lumens and the bronchial lumen as measured using the profile projector in the 35 and 37 Fr tubes are listed in Table 6. Cross-sectional views of each tube are shown in Fig. 6. We found large disparities between each manufacturer’s tubes. Both lumen parts in all tubes were longer in the horizontal measurement (Fig. 6).

**Table 6:** Measuremetn of the external diameters of both lumens and the tracheal and bronchial cuff inflation tubules (Unit: mm)

Product Name	**35Fr**																
	**Sample Number**	**Lot No**	**External Diameter (Long and Short Axes) of Both Lumens Part**			**Transverse and Longitudinal Diameters of Bronchial Lumen Part**			**Transverse and Longitudinal Diameters of Tubules for Tracheal Cuff Inflation at both Lumens Part**			**Transverse and Longitudinal Diameters of Tubules for Bronchial Cuff Inflation at both Lumens Part**			**Transverse and Longitudinal Diameters of Tubules for Bronchial Cuff Inflation at Bronchial Lumen Part**		
			**Long**	**Short**	**Mean**	**Transverse**	**Longitudinal**	**Mean**	**Transverse**	**Longitudinal**	**Mean**	**Transverse**	**Longitudinal**	**Mean**	**Transverse**	**Longitudinal**	**Mean**
Bronch-Cath™	①	201210608X	13.0	11.9	12.45	9.1	9.9	9.50	0.52	1.08	0.800	0.53	1.12	0.825	0.49	1.31	0.900
	②	201503168X	13.2	12.1	12.65	8.9	10.9	9.90	0.60	1.10	0.850	0.57	1.08	0.825	0.53	1.25	0.890
		Mean	13.10	12.00	12.55	9.00	10.40	9.70	0.56	1.09	0.825	0.55	1.10	0.825	0.51	1.28	0.895
Blue Line™	③	3227850	12.8	10.9	11.85	6.9	10.5	8.70	0.56	1.13	0.845	0.60	1.16	0.880	0.59	1.16	0.875
	④	3227851	13.0	11.0	12.00	7.7	10.4	9.05	0.59	1.18	0.885	0.65	1.21	0.930	0.62	1.20	0.910
		Mean	12.90	10.95	11.93	7.30	10.45	8.88	0.58	1.16	0.865	0.63	1.19	0.905	0.61	1.18	0.893
Bronchopart®	⑤	15GE29J	12.6	10.8	11.70	9.0	10.1	9.55	0.54	0.57	0.555	0.38	0.64	0.510	0.48	0.75	0.615
	⑥	15DE16J	12.5	10.9	11.70	8.6	9.9	9.25	0.43	0.62	0.525	0.40	0.57	0.485	0.44	0.75	0.595
		Mean	12.55	10.85	11.70	8.80	10.00	9.40	0.49	0.60	0.540	0.39	0.61	0.498	0.46	0.75	0.605
SHER-I-BRONCH®	⑦	73L1500302	13.4	11.1	12.25	8.2	11.3	9.75	0.80	0.55	0.675	0.78	0.47	0.625	0.98	0.70	0.840
	⑧	73L1500168	13.2	11.5	12.35	8.3	11.1	9.70	0.89	0.79	0.840	0.73	0.63	0.680	0.80	0.75	0.775
		Mean	13.3	11.3	12.30	8.3	11.2	9.73	0.85	0.67	0.758	0.76	0.55	0.653	0.89	0.73	0.808
Coopdech	⑨	B161013X	12.6	10.7	11.65	8.3	8.3	8.30	1.23	1.29	1.260	1.14	1.06	1.100	0.97	0.98	0.975
	⑩	B161017X	12.6	10.7	11.65	8.3	8.4	8.35	1.29	1.31	1.300	1.31	1.34	1.325	0.95	0.99	0.970
		Mean	12.6	10.7	11.65	8.3	8.4	8.33	1.26	1.30	1.280	1.23	1.20	1.213	0.96	0.99	0.973
	**37Fr**																
	**Sample Number**	**Lot No**	**External Diameter (Long and Short Axes) of Both Lumens Part**			**Transverse and Longitudinal Diameters of Bronchial Lumen Part**			**Transverse and Longitudinal Diameters of Tubules for Tracheal Cuff Inflation at both Lumens Part**			**Transverse and Longitudinal Diameters of Tubules for Bronchial Cuff Inflation at both Lumens Part**			**Transverse and Longitudinal Diameters of Tubules for Bronchial Cuff Inflation at Bronchial Lumen Part**		
			**Long**	**Short**	**Mean**	**Transverse**	**Longitudinal**	**Mean**	**Transverse**	**Longitudinal**	**Mean**	**Transverse**	**Longitudinal**	**Mean**	**Transverse**	**Longitudinal**	**Mean**
	⑪	201411237X	13.8	13.1	13.45	9.5	11.2	10.35	0.64	1.10	0.870	0.50	1.25	0.875	0.52	1.49	1.005
	⑫	201410348X	13.7	12.7	13.20	9.5	11.2	10.35	0.63	1.20	0.915	0.61	1.26	0.935	0.57	1.48	1.025
		Mean	13.75	12.90	13.33	9.50	11.20	10.35	0.64	1.15	0.893	0.56	1.26	0.905	0.55	1.49	1.015
	⑬	3227862	13.4	11.3	12.35	7.3	10.5	8.90	0.58	1.13	0.855	0.61	1.11	0.860	0.60	1.09	0.845
	⑭	3227865	13.3	11.3	12.30	7.7	10.7	9.20	0.63	1.09	0.860	0.58	1.05	0.815	0.59	1.12	0.855
		Mean	13.35	11.30	12.33	7.50	10.60	9.05	0.61	1.11	0.858	0.60	1.08	0.838	0.60	1.11	0.850
	⑮	16HE33J	13.1	11.3	12.20	9.5	10.2	9.85	0.56	0.70	0.630	0.42	0.78	0.600	0.46	0.93	0.695
	⑯	15GE29J	13.4	11.4	12.40	9.7	10.6	10.15	0.45	0.74	0.595	0.49	0.61	0.550	0.43	0.81	0.620
		Mean	13.25	11.35	12.30	9.60	10.40	10.00	0.51	0.72	0.613	0.46	0.70	0.575	0.45	0.87	0.658
	⑰	73L1500369	14.2	11.8	13.00	8.8	11.7	10.25	0.73	0.81	0.770	0.71	0.73	0.720	0.73	0.84	0.785
	⑱	73K1600076	14.0	12.1	13.05	8.6	11.3	9.95	0.82	0.81	0.815	0.80	0.78	0.790	0.85	0.83	0.840
		Mean	14.10	11.95	13.03	8.70	11.50	10.10	0.78	0.81	0.793	0.76	0.76	0.755	0.79	0.84	0.813
	⑲	B161205X	13.7	11.2	12.45	8.5	8.5	8.50	1.28	1.29	1.285	1.30	1.29	1.295	1.02	0.96	0.990
	⑳	B161209X	13.7	11.2	12.45	8.5	8.6	8.55	1.32	1.30	1.310	1.26	1.26	1.260	0.98	1.00	0.990
		Mean	13.70	11.20	12.45	8.50	8.55	8.53	1.30	1.30	1.298	1.28	1.28	1.278	1.00	0.98	0.990

X-X: long axes; Y-Y: short axes

In the SHER-I-BRONCH®, the tubule for bronchial cuff inflation was at the upper left and that for tracheal cuff inflation was at the lower right

### Measurement of various cross-sectional areas

As shown in Table 7, we found large disparities in the various cross-sectional areas measured between each manufacturer’s tubes. The cross-sectional areas of the tracheal and bronchial lumens were similar in all of the tubes.

**Table 7:** Measurement of various cross-sectional areas (Unit of Area: mm.^2^)

**Size**			**Both Lumens Part**				**Bronchial Lumen Part**	
	**Product Name**	**Sample Number**	**Tracheal Lumen**	**Bronchial Lumen**	**Tubule for Tracheal Cuff Inflation**	**Tubule for Bronchial Cuff Inflation**	**Bronchial Lumen**	**Tubule for Bronchial Cuff Inflation**
**35Fr**	Bronch-Cath™	①	26.23	25.91	0.448	0.467	35.45	0.532
		②	27.86	27.81	0.411	0.486	35.80	0.525
		Mean	27.045	26.860	0.4295	0.4765	35.625	0.5285
	Blue Line™	③	24.22	25.20	0.536	0.576	25.17	0.545
		④	24.92	26.24	0.514	0.498	27.35	0.558
		Mean	24.570	25.720	0.5250	0.5370	26.260	0.5515
	Bronchopart®	⑤	22.00	21.40	0.183	0.194	33.23	0.237
		⑥	23.05	23.98	0.179	0.225	32.31	0.240
		Mean	22.525	22.690	0.1810	0.2095	32.770	0.2385
	SHER-I-BRONCH®	⑦	31.97	32.99	0.477	0.480	38.71	0.494
		⑧	30.62	32.59	0.306	0.445	39.11	0.497
		Mean	31.295	32.790	0.3915	0.4625	38.910	0.4955
	Coopdech	⑨	24.61	24.24	1.161	1.230	17.26	0.729
		⑩	27.01	26.90	1.359	1.340	18.70	0.728
		Mean	25.810	25.570	1.2600	1.2850	17.980	0.7285
**37Fr**	Bronch-Cath™	⑪	35.51	35.31	0.588	0.518	47.78	0.686
		⑫	33.11	33.17	0.453	0.574	48.30	0.826
		Mean	34.310	34.240	0.5205	0.5460	48.040	0.7560
	Blue Line™	⑬	29.67	31.70	0.398	0.424	30.04	0.511
		⑭	30.73	32.59	0.540	0.515	30.92	0.536
		Mean	30.200	32.145	0.4690	0.4695	30.480	0.5235
	Bronchopart®	⑮	24.96	24.06	0.221	0.271	38.26	0.331
		⑯	27.28	28.13	0.234	0.294	43.22	0.328
		Mean	26.120	26.095	0.2275	0.2825	40.740	0.3295
	SHER-I-BRONCH®	⑰	31.32	32.51	0.384	0.426	39.38	0.405
		⑱	32.43	33.46	0.408	0.421	39.66	0.281
		Mean	31.875	32.985	0.3960	0.4235	39.520	0.3430
	Coopdech	⑲	30.96	30.64	1.211	1.207	19.46	0.659
		⑳	30.75	30.47	1.184	1.222	19.77	0.719
		Mean	30.855	30.555	1.1975	1.2145	19.615	0.6890

## Discussion

The present study showed large disparities in the measurements performed in our nine investigations of five different manufacturers’ DLTs. Our findings satisfied the original hypothesis that large disparities in terms of inaccurate dimensions and potential disadvantages might exist between DLTs of different manufacturers.

### Passage of steel balls of various sizes

We attempted to pass steel balls of various sizes through the DLTs to simulate the ease of passing a bronchofiberscope or a suction catheter. We believe that using steel balls is one objective method as a methodology: the cross-section of bronchofiberscopes is a round shape, and therefore, the size of the inscribed circle of the tube lumen affects the passage of the bronchofiberscope. Thus, we could easily confirm the size of the inscribed circle of the overall tube lumen. The reason for performing two trials for each ball was because the material of DLTS is limber, the section of the lumens can change slightly with tube position and other movements, and therefore, passage of the steel balls through the lumen can be uneven.

The 35 Fr Bronchopart® was inferior because a 4.0-mm steel ball could not pass through its tracheal lumen. Likewise. 4.0-mm steel balls could not pass through the bronchial lumens of one Blue Line™ tube and one Bronchopart® tube, indicating these tubes to be inferior for their bronchial lumens. The SHER-I-BRONCH® was judged the best tube in this study because a 4.5-mm steel ball could pass through it. Among the 37 Fr tubes, the Bronch-Cath™ and Coopdech were considered superior because 4.5-mm steel balls passed through the tracheal lumen of both DLTs. In the bronchial lumen of the 37 Fr tubes, 4.5-mm steel balls only passed through the SHER-I-BRONCH® and Coopdech, and these DLTs were considered superior for their bronchial lumens. Overall, we considered the Coopdech to be the best 37 Fr tube in this study (Table 2).

### Measurement of the external diameters of tracheal and bronchial cuffs

Choosing the correct size of a left DLT for an individual patient is important. An inappropriately small DLT will either fail to provide lung isolation or will require endobronchial cuff volumes and pressures that could damage the bronchus, whereas too large a DLT can damage the trachea or bronchus [9]. Determining appropriate tube size is difficult as the left main bronchus internal diameter does not correlate closely with sex, age, height, or weight, and only moderately correlates with tracheal size [10]. Although Slinger [11] recommended radiological measurement of the left mainstem bronchial diameter as an objective guide to choosing a left DLT size, measurement of left bronchial diameter on chest computed tomographic scans can objectively guide the choice of left DLT size for an individual patient [9]. Hegland et al. [3] also measured outer cuff diameters in their recent publication, but they did not obtain transverse and longitudinal measurements, despite the fact that cuffs may not be truly round in configuration. This was our justification for including these dimensions in our study.

We found discrepancies between the external diameters of the different manufacturers’ cuffs in both the 35 and 37 Fr tubes. Furthermore, the external diameter of the 37 Fr Blue Line™ bronchial cuff was smaller than that of the 35 Fr Blue Line™ bronchial cuff, and the 37 Fr Bronchopart® bronchial cuff was also smaller than the 35 Fr Bronchopart.® bronchial cuff (Table 3). Practitioners should be aware of the external diameters of the tracheal and bronchial cuffs (Table 3) and the external diameters of both the lumen part and the bronchial lumen part (Table 6; Study V) to avoid failing to provide adequate lung isolation and to prevent complications such as sore throat, tracheal mucosa ulcers, tracheal rupture, and subglottic stenosis, etc. [12].

### Measurement of length of the cuff and tip of the bronchial segment of the tubes

One of the most important bronchial segments is “A = (a + b)” (Fig. 1) because of its involvement in the margin of safety [2, 5]. The margin of safety for a DLT is the length of the tracheobronchial tree between the most distal and proximal acceptable positions [13]. If the length of the cuff plus the tip exceeds that of the left main bronchus, the left upper lobe bronchus will be occluded [2]. Benumof et al. [5] concluded that the bevel of the left lumen tip of a left-sided tube should be made shorter and closer to the left endobronchial cuff, and the left lumen cuff should be narrower. The 35 and 37 Fr Bronchopart® tubes were considered the most advantageous because “A” was the shortest in these tubes (Table 4).

In 2006, Partridge et al. [2] measured bronchial cuff and bronchial tip lengths on 220 used left DLTs from four manufacturers. The largest cuff-tip length (40 mm) was found in the Portex (Blue Line™) 41 Fr tube, but the cuff-tip lengths of some 41 Fr tubes from each manufacturer were 33 mm or greater, longer than the shortest left main bronchus as measured by Benumof et al. [5] With the shortest cuff-tip length of the DLTs examined, the Rüsch (Bronchopart®) would appear to be advantageous. Marked variations were found in the cuff-tip lengths of tubes of the same size from the same manufacturer, with the largest variation (18 mm) found in the Portex 41 Fr tube. At least one French size of each of the manufacturers’ DLTs showed substantial variation of 8 mm or more. Partridge et al. concluded that users must understand that cuff-tip length can vary significantly, and they must match the chosen tube to the patient to preserve an adequate margin of safety. The results of the study of Partridge et al. were similar with ours (Fig. 1, Table 4).

### Measurement of various lengths of the tubes

We measured four different lengths of the tubes. We considered that length “II” strongly relates to ease-of-use factors such as handling tendency, bronchoscope or suction tube insertion, and how far a suction catheter will reach. Although the COVIDIEN catalog [8] shows the Bronch-Cath™ length to be 420 mm (35 and 37 Fr), our measurements were 436.5 mm (35 Fr) and 438.5 mm (37 Fr) (Table 5), and disparities were present among all products investigated. We emphasize that the lengths of IV of the Bronchopart® were 295.0 mm (35 Fr) and 299.5 mm (37 Fr) (Table 5), indicating that the very hard Y-shaped connector could enter the patient’s oral cavity. Users must be aware of these variances in the lengths of different manufacturers’ DLTs, which can affect resistance to flow and maneuverability of an endotracheal suction catheter or fiberoptic bronchoscope.

### Measurement of the external diameters of both lumens and the tracheal and bronchial cuff inflation tubules

Russell et al. [10] manually measured bronchial tube diameter in 171 left DLTs ranging from nominal French gauge 28 to 41 from four manufacturers. We, however, measured these diameters using a profile projector. One reason the results of Russell et al. are not comparable with ours is that they manually measured used tubes. Nevertheless, they found a substantial overlap between sizes, even from the same manufacturer, and that the tubes did not correspond to their stated French gauge at the bronchial segment level, all being much smaller than nominal size [10]. We found no substantial overlap between the diameters of the bronchial lumen segment owing to improvements of tube quality but also to our small sample numbers.

### Measurement of various cross-sectional areas

Hegland et al. [3] recently measured the cross-sectional area of the DLTs utilizing the measured width and height of the tube according to the formula “cross-sectional area = π × width/2 (= lateral) × height/2 (= anterior–posterior), whereas we measured various cross-sectional areas of tracheal and bronchial “lumens” and “tubules” for cuff inflation with area measurement software.

Some tubes had especially small cross-sectional areas along with substantial deformation of the lumens and tubules, and we also found disparities between the different lots except for the Coopdech (Fig. 6). The measured cross-sectional areas corresponded with the difficulty in passing the steel balls. The 35 Fr Bronchopart® was inferior for its tracheal lumen size (Table 2) and, in fact, the cross-sectional areas of this tube’s tracheal and bronchial lumens were the smallest (Table 7). Furthermore, the 35 Fr SHER-I-BRONCH® was the best tube in the steel ball experiment (Table 2), and the cross-sectional areas of the tracheal and bronchial lumens and the bronchial lumen in this tube were indeed the largest (Table 7). Similarly, in accordance with the findings in the steel ball experiment, the 37 Fr Bronchopart®, which was inferior for both lumens, showed the smallest cross-sectional areas of the tracheal and bronchial lumens (Table 7).

The tubules in both lumen parts were smallest in the Bronchopart® (about 0.2–0.3 mm^2^) and largest in Coopdech (about 1.2–1.3 mm^2^) (Table 7), indicating potentially easier and faster cuff inflation or deflation in the Coopdech.

We believe that it is almost impossible to measure the inner dimensions of the tubes because the inner part of the tubes is not a circle (Fig. 6). Therefore, we conducted the “Passage of steel balls of various sizes” and “Measurement of various cross-sectional areas” studies because especially, the “Passage of steel balls of various sizes” study could detect the narrowest size of the inner part of each tube.

### Study limitations

This study has several limitations. We only obtained two different lot numbers of each tube type. The results might differ if greater numbers of different lots were examined. Furthermore, in fact, we could obtain neither all manufacturer DLTs nor all sizes in the market because of research funds and limited time and situation, etc. (e.g., Daiken Medical sold only 35 and 37 Fr tubes when we conducted this research). However, we believe that to examine our hypothesis, our method was not incorrect to discover disparities between each manufacturer’s tubes because these tubes are “industrial products”. As this may be a limitation of this study, in the future, as a next step, all manufacturers’ DLTs and all sizes in the market might be investigated. Second, although we tried to pass steel balls of various sizes through the tubes to simulate the ease of passing a bronchofiberscope and suction catheters by gravity, this is not the same as attempting passage with a real bronchofiberscope and suction catheters because they are sometimes lubricated in the clinical setting, and also, clinically, DLTs adopt the anatomical shape of the curvature beginning from the oropharynx to the proximal primary bronchi. This is evident by the increased resistance to passage of a bronchofiberscope and suction catheter experienced along the segment of increased curvature from the oropharynx to larynx depending on neck flexure and positioning. Nonetheless, we could find disparities between each manufacturer’s tubes. It would be ideal to compare our results with the resistance experienced with many different bronchofiberscopes on the market throughout the world, and thus, further study is needed.

## Conclusions

This study was a technical assessment of various features and aspects of DLTs from different manufacturers. It raises awareness that there can be important differences in sizing between manufacturers that could potentially be clinically relevant as product labeling and specification sheets lack details that might affect selection of a specific tube size.

Our findings suggest that we might change the manner in which we select DLT tube sizes or tube manufacturers to avoid unexpected trouble and complications by especially considering the following results. We would like to emphatically emphasize that practitioners should know i) the external diameters of the cuffs (Table 3; second study), and the external diameters of both the lumen part and the bronchial lumen part (Table 6; fifth study) to avoid failing to provide lung isolation and avoiding complications such as sore throat, tracheal mucosa ulcers, tracheal rupture, and subglottic stenosis, etc. [12] ii) the margin of safety (Fig. 1, Table 4; third study), and iii) the length of “IV” of the Bronchopart® (Table 5; fourth study), which indicates the potential for the very hard Y-shaped connector to enter the patient’s oral cavity.

Moreover, we found large disparities between each manufacturer’s tubes in our six investigations and three supplemental studies, but these disparities may be due to different lots or changes in specifications made by each manufacturer. Therefore, we consider that the present results do not per se indicate good or bad performance and/or overall tube quality, but there are advantages and disadvantages of each product. Nevertheless, we found tubes exhibiting lower quality, such as deformations, and non-universal and inconsistent sizing, in the comparison of the manufacturers’ tubes. Practitioners should be aware of the features and aspects and the differences of these tubes. The present study itself is important in that it raises questions about quality control of DLTs at the manufacturer level.

## Supplementary Information

**Additional file 1.**

**Additional file 2.**

**Additional file 3.**

## Data Availability

The datasets used and/or analyzed during the current study are available from the corresponding author on reasonable request.
