# Supplementary material for: Comparisons of the segments of left-sided double-lumen tracheobronchial tubes as industrial products
Source: BMC Anesthesiol. 2022 Jun 8;22:177. doi: 10.1186/s12871-022-01698-2 (PMC9175383; doi:10.1186/s12871-022-01698-2)
Supplement: Supplementary file 1 — Additional file 1. [file 12871_2022_1698_MOESM1_ESM.pptx]

## Slide 1
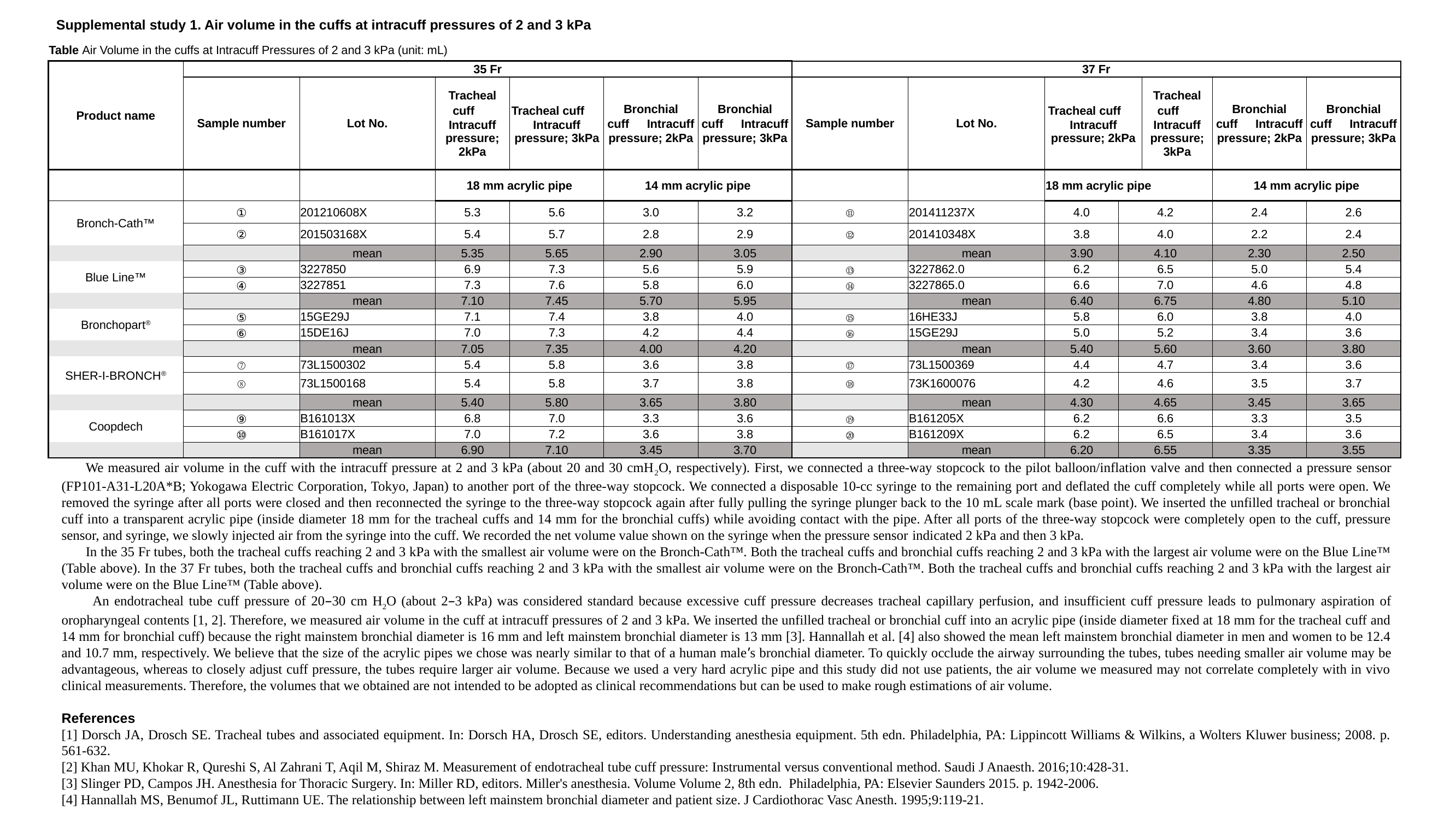

Supplemental study 1. Air volume in the cuffs at intracuff pressures of 2 and 3 kPa
| Table Air Volume in the cuffs at Intracuff Pressures of 2 and 3 kPa (unit: mL) | | | | | | | | | | | | | | |
| --- | --- | --- | --- | --- | --- | --- | --- | --- | --- | --- | --- | --- | --- | --- |
| Product name | 35 Fr | | | | | | 37 Fr | | | | | | | |
| | Sample number | Lot No. | Tracheal cuff　Intracuff pressure; 2kPa | Tracheal cuff　Intracuff pressure; 3kPa | Bronchial cuff　Intracuff pressure; 2kPa | Bronchial cuff　Intracuff pressure; 3kPa | Sample number | Lot No. | Tracheal cuff　Intracuff pressure; 2kPa | Tracheal cuff　Intracuff pressure; 2kPa | Tracheal cuff　Intracuff pressure; 3kPa | Tracheal cuff　Intracuff pressure; 3kPa | Bronchial cuff　Intracuff pressure; 2kPa | Bronchial cuff　Intracuff pressure; 3kPa |
| | | | 18 mm acrylic pipe | | 14 mm acrylic pipe | | | | 18 mm acrylic pipe | 18 mm acrylic pipe | | | 14 mm acrylic pipe | |
| Bronch-Cath™ | ① | 201210608X | 5.3 | 5.6 | 3.0 | 3.2 | ⑪ | 201411237X | 4.0 | 4.0 | 4.2 | | 2.4 | 2.6 |
| | ② | 201503168X | 5.4 | 5.7 | 2.8 | 2.9 | ⑫ | 201410348X | 3.8 | 3.8 | 4.0 | | 2.2 | 2.4 |
| | | mean | 5.35 | 5.65 | 2.90 | 3.05 | | mean | 3.90 | 3.90 | 4.10 | | 2.30 | 2.50 |
| Blue Line™ | ③ | 3227850 | 6.9 | 7.3 | 5.6 | 5.9 | ⑬ | 3227862.0 | 6.2 | 6.2 | 6.5 | | 5.0 | 5.4 |
| | ④ | 3227851 | 7.3 | 7.6 | 5.8 | 6.0 | ⑭ | 3227865.0 | 6.6 | 6.6 | 7.0 | | 4.6 | 4.8 |
| | | mean | 7.10 | 7.45 | 5.70 | 5.95 | | mean | 6.40 | 6.40 | 6.75 | | 4.80 | 5.10 |
| Bronchopart® | ⑤ | 15GE29J | 7.1 | 7.4 | 3.8 | 4.0 | ⑮ | 16HE33J | 5.8 | 5.8 | 6.0 | | 3.8 | 4.0 |
| | ⑥ | 15DE16J | 7.0 | 7.3 | 4.2 | 4.4 | ⑯ | 15GE29J | 5.0 | 5.0 | 5.2 | | 3.4 | 3.6 |
| | | mean | 7.05 | 7.35 | 4.00 | 4.20 | | mean | 5.40 | 5.40 | 5.60 | | 3.60 | 3.80 |
| SHER-I-BRONCH® | ⑦ | 73L1500302 | 5.4 | 5.8 | 3.6 | 3.8 | ⑰ | 73L1500369 | 4.4 | 4.4 | 4.7 | | 3.4 | 3.6 |
| | ⑧ | 73L1500168 | 5.4 | 5.8 | 3.7 | 3.8 | ⑱ | 73K1600076 | 4.2 | 4.2 | 4.6 | | 3.5 | 3.7 |
| | | mean | 5.40 | 5.80 | 3.65 | 3.80 | | mean | 4.30 | 4.30 | 4.65 | | 3.45 | 3.65 |
| Coopdech | ⑨ | B161013X | 6.8 | 7.0 | 3.3 | 3.6 | ⑲ | B161205X | 6.2 | 6.2 | 6.6 | | 3.3 | 3.5 |
| | ⑩ | B161017X | 7.0 | 7.2 | 3.6 | 3.8 | ⑳ | B161209X | 6.2 | 6.2 | 6.5 | | 3.4 | 3.6 |
| | | mean | 6.90 | 7.10 | 3.45 | 3.70 | | mean | 6.20 | 6.20 | 6.55 | | 3.35 | 3.55 |
We measured air volume in the cuff with the intracuff pressure at 2 and 3 kPa (about 20 and 30 cmH2O, respectively). First, we connected a three-way stopcock to the pilot balloon/inflation valve and then connected a pressure sensor (FP101-A31-L20A*B; Yokogawa Electric Corporation, Tokyo, Japan) to another port of the three-way stopcock. We connected a disposable 10-cc syringe to the remaining port and deflated the cuff completely while all ports were open. We removed the syringe after all ports were closed and then reconnected the syringe to the three-way stopcock again after fully pulling the syringe plunger back to the 10 mL scale mark (base point). We inserted the unfilled tracheal or bronchial cuff into a transparent acrylic pipe (inside diameter 18 mm for the tracheal cuffs and 14 mm for the bronchial cuffs) while avoiding contact with the pipe. After all ports of the three-way stopcock were completely open to the cuff, pressure sensor, and syringe, we slowly injected air from the syringe into the cuff. We recorded the net volume value shown on the syringe when the pressure sensor indicated 2 kPa and then 3 kPa.
In the 35 Fr tubes, both the tracheal cuffs reaching 2 and 3 kPa with the smallest air volume were on the Bronch-Cath™. Both the tracheal cuffs and bronchial cuffs reaching 2 and 3 kPa with the largest air volume were on the Blue Line™ (Table above). In the 37 Fr tubes, both the tracheal cuffs and bronchial cuffs reaching 2 and 3 kPa with the smallest air volume were on the Bronch-Cath™. Both the tracheal cuffs and bronchial cuffs reaching 2 and 3 kPa with the largest air volume were on the Blue Line™ (Table above).
 An endotracheal tube cuff pressure of 20–30 cm H2O (about 2–3 kPa) was considered standard because excessive cuff pressure decreases tracheal capillary perfusion, and insufficient cuff pressure leads to pulmonary aspiration of oropharyngeal contents [1, 2]. Therefore, we measured air volume in the cuff at intracuff pressures of 2 and 3 kPa. We inserted the unfilled tracheal or bronchial cuff into an acrylic pipe (inside diameter fixed at 18 mm for the tracheal cuff and 14 mm for bronchial cuff) because the right mainstem bronchial diameter is 16 mm and left mainstem bronchial diameter is 13 mm [3]. Hannallah et al. [4] also showed the mean left mainstem bronchial diameter in men and women to be 12.4 and 10.7 mm, respectively. We believe that the size of the acrylic pipes we chose was nearly similar to that of a human male’s bronchial diameter. To quickly occlude the airway surrounding the tubes, tubes needing smaller air volume may be advantageous, whereas to closely adjust cuff pressure, the tubes require larger air volume. Because we used a very hard acrylic pipe and this study did not use patients, the air volume we measured may not correlate completely with in vivo clinical measurements. Therefore, the volumes that we obtained are not intended to be adopted as clinical recommendations but can be used to make rough estimations of air volume.
References
[1] Dorsch JA, Drosch SE. Tracheal tubes and associated equipment. In: Dorsch HA, Drosch SE, editors. Understanding anesthesia equipment. 5th edn. Philadelphia, PA: Lippincott Williams & Wilkins, a Wolters Kluwer business; 2008. p. 561-632.
[2] Khan MU, Khokar R, Qureshi S, Al Zahrani T, Aqil M, Shiraz M. Measurement of endotracheal tube cuff pressure: Instrumental versus conventional method. Saudi J Anaesth. 2016;10:428-31.
[3] Slinger PD, Campos JH. Anesthesia for Thoracic Surgery. In: Miller RD, editors. Miller's anesthesia. Volume Volume 2, 8th edn. Philadelphia, PA: Elsevier Saunders 2015. p. 1942-2006.
[4] Hannallah MS, Benumof JL, Ruttimann UE. The relationship between left mainstem bronchial diameter and patient size. J Cardiothorac Vasc Anesth. 1995;9:119-21.
