# Supplementary material for: Comparisons of the segments of left-sided double-lumen tracheobronchial tubes as industrial products
Source: BMC Anesthesiol. 2022 Jun 8;22:177. doi: 10.1186/s12871-022-01698-2 (PMC9175383; doi:10.1186/s12871-022-01698-2)
Supplement: Supplementary file 2 — Additional file 2. [file 12871_2022_1698_MOESM2_ESM.pptx]

## Slide 1
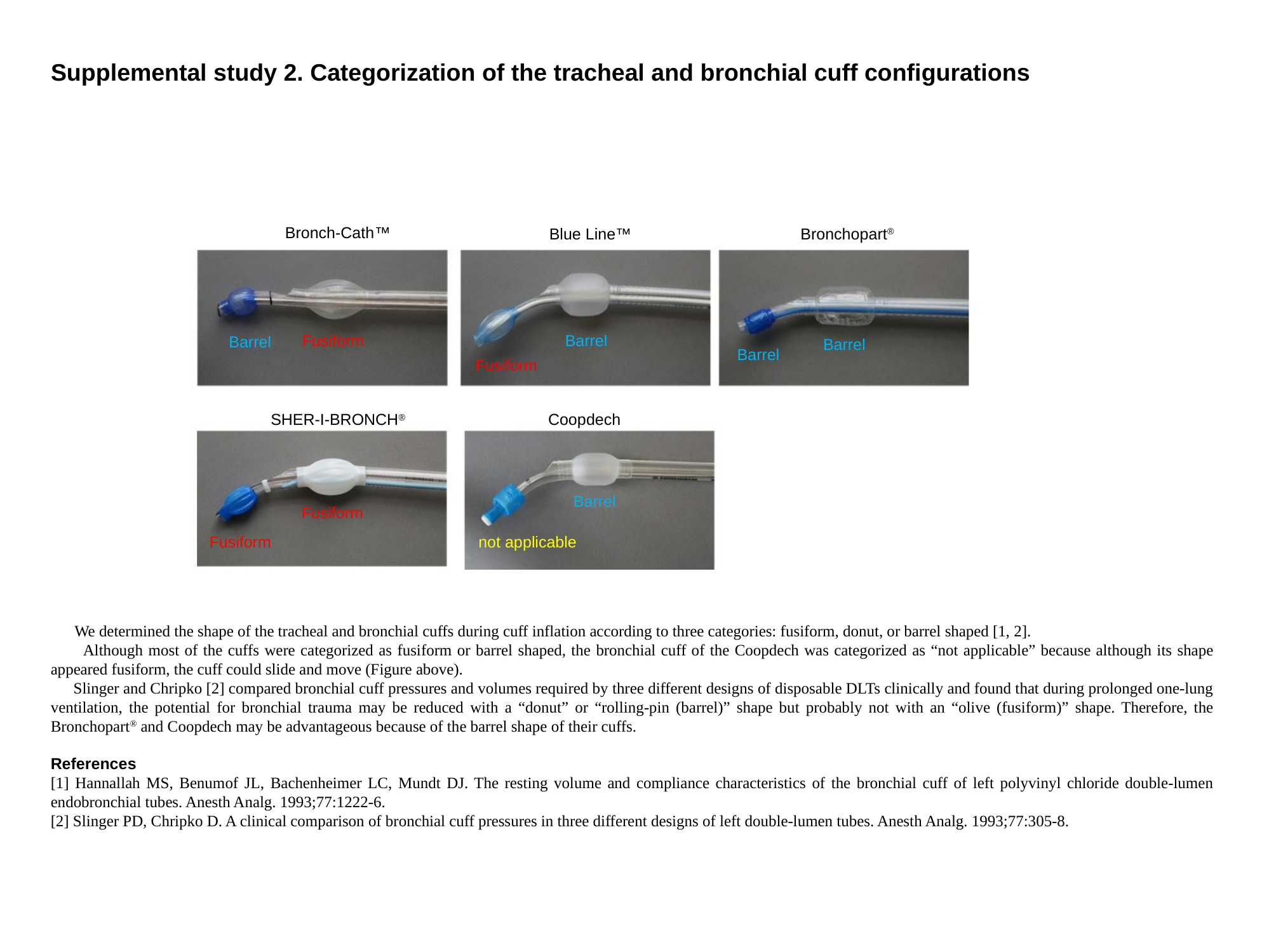

# Supplemental study 2. Categorization of the tracheal and bronchial cuff configurations
Bronch-Cath™
Blue Line™
Bronchopart®
Fusiform
Barrel
Barrel
Barrel
Barrel
Fusiform
Coopdech
SHER-I-BRONCH®
Barrel
Fusiform
not applicable
Fusiform
　 We determined the shape of the tracheal and bronchial cuffs during cuff inflation according to three categories: fusiform, donut, or barrel shaped [1, 2].
 Although most of the cuffs were categorized as fusiform or barrel shaped, the bronchial cuff of the Coopdech was categorized as “not applicable” because although its shape appeared fusiform, the cuff could slide and move (Figure above).
 Slinger and Chripko [2] compared bronchial cuff pressures and volumes required by three different designs of disposable DLTs clinically and found that during prolonged one-lung ventilation, the potential for bronchial trauma may be reduced with a “donut” or “rolling-pin (barrel)” shape but probably not with an “olive (fusiform)” shape. Therefore, the Bronchopart® and Coopdech may be advantageous because of the barrel shape of their cuffs.
References
[1] Hannallah MS, Benumof JL, Bachenheimer LC, Mundt DJ. The resting volume and compliance characteristics of the bronchial cuff of left polyvinyl chloride double-lumen endobronchial tubes. Anesth Analg. 1993;77:1222-6.
[2] Slinger PD, Chripko D. A clinical comparison of bronchial cuff pressures in three different designs of left double-lumen tubes. Anesth Analg. 1993;77:305-8.
