# Supplementary material for: Comparisons of the segments of left-sided double-lumen tracheobronchial tubes as industrial products
Source: BMC Anesthesiol. 2022 Jun 8;22:177. doi: 10.1186/s12871-022-01698-2 (PMC9175383; doi:10.1186/s12871-022-01698-2)
Supplement: Supplementary file 3 — Additional file 3. [file 12871_2022_1698_MOESM3_ESM.pptx]

## Slide 1
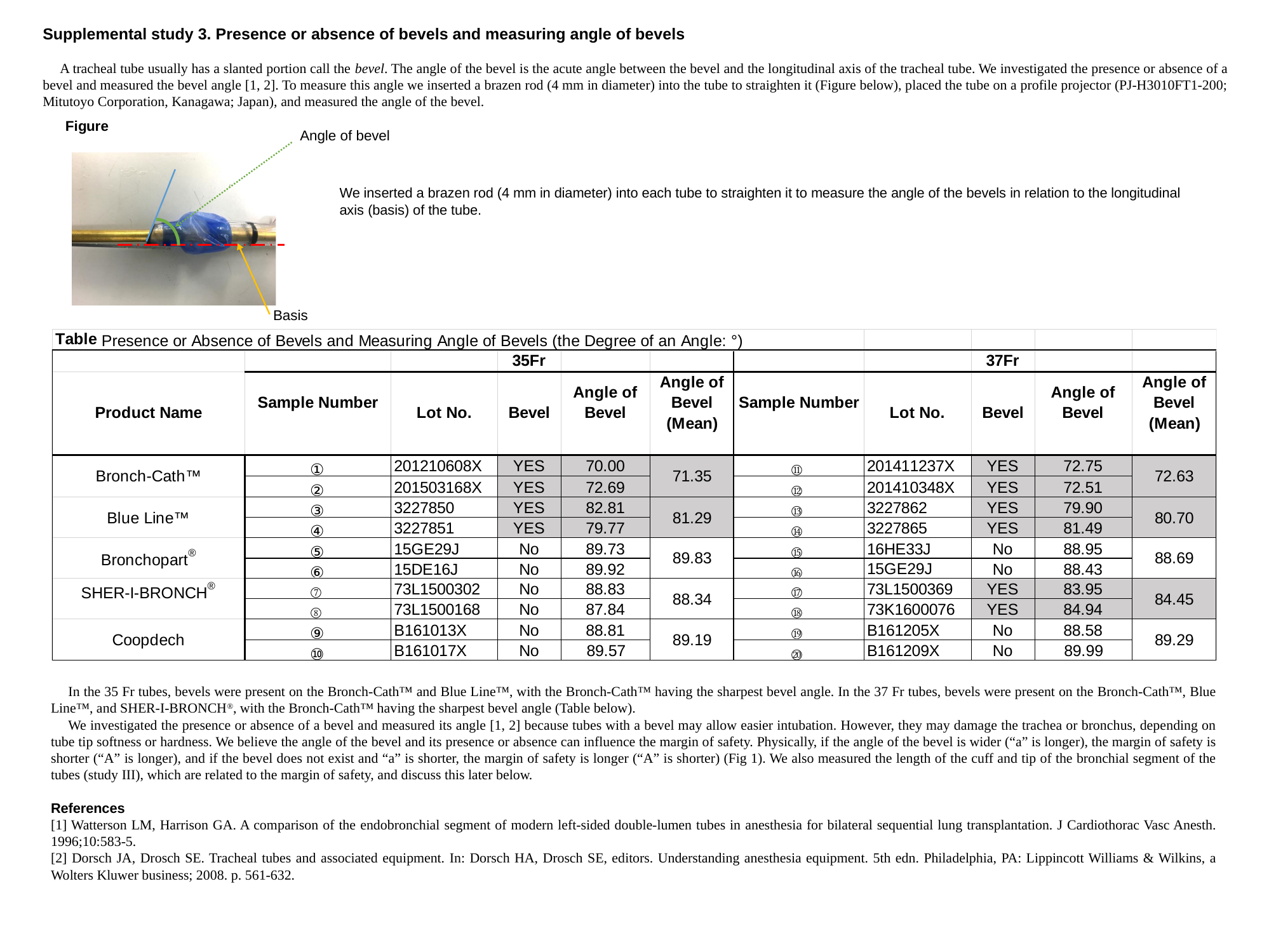

Supplemental study 3. Presence or absence of bevels and measuring angle of bevels
　A tracheal tube usually has a slanted portion call the bevel. The angle of the bevel is the acute angle between the bevel and the longitudinal axis of the tracheal tube. We investigated the presence or absence of a bevel and measured the bevel angle [1, 2]. To measure this angle we inserted a brazen rod (4 mm in diameter) into the tube to straighten it (Figure below), placed the tube on a profile projector (PJ-H3010FT1-200; Mitutoyo Corporation, Kanagawa; Japan), and measured the angle of the bevel.
We inserted a brazen rod (4 mm in diameter) into each tube to straighten it to measure the angle of the bevels in relation to the longitudinal axis (basis) of the tube.
　In the 35 Fr tubes, bevels were present on the Bronch-Cath™ and Blue Line™, with the Bronch-Cath™ having the sharpest bevel angle. In the 37 Fr tubes, bevels were present on the Bronch-Cath™, Blue Line™, and SHER-I-BRONCH®, with the Bronch-Cath™ having the sharpest bevel angle (Table below).
　We investigated the presence or absence of a bevel and measured its angle [1, 2] because tubes with a bevel may allow easier intubation. However, they may damage the trachea or bronchus, depending on tube tip softness or hardness. We believe the angle of the bevel and its presence or absence can influence the margin of safety. Physically, if the angle of the bevel is wider (“a” is longer), the margin of safety is shorter (“A” is longer), and if the bevel does not exist and “a” is shorter, the margin of safety is longer (“A” is shorter) (Fig 1). We also measured the length of the cuff and tip of the bronchial segment of the tubes (study III), which are related to the margin of safety, and discuss this later below.
References
[1] Watterson LM, Harrison GA. A comparison of the endobronchial segment of modern left-sided double-lumen tubes in anesthesia for bilateral sequential lung transplantation. J Cardiothorac Vasc Anesth. 1996;10:583-5.
[2] Dorsch JA, Drosch SE. Tracheal tubes and associated equipment. In: Dorsch HA, Drosch SE, editors. Understanding anesthesia equipment. 5th edn. Philadelphia, PA: Lippincott Williams & Wilkins, a Wolters Kluwer business; 2008. p. 561-632.
